# Deformation characteristics of solid-state benzene as a step towards understanding planetary geology

**DOI:** 10.1038/s41467-022-35647-x

**Published:** 2022-12-26

**Authors:** Wenxin Zhang, Xuan Zhang, Bryce W. Edwards, Lei Zhong, Huajian Gao, Michael J. Malaska, Robert Hodyss, Julia R. Greer

**Affiliations:** 1 Division of Engineering and Applied Sciences, California Institute of Technology, 1200 E. California Blvd, Pasadena, CA 91125 USA; 2 INM—Leibniz Institute for New Materials, Campus D2 2, 66123 Saarbrücken, Germany; 3 School of Engineering, Brown University, Providence, RI 02912 USA; 4 School of Mechanical and Aerospace Engineering, College of Engineering, Nanyang Technological University, 70 Nanyang Drive, 639798 Singapore, Singapore; 5 Institute of High Performance Computing, A*STAR, 138632 Singapore, Singapore; 6 Jet Propulsion Laboratory, California Institute of Technology, 4800 Oak Grove Drive, Pasadena, CA 91109 USA; 7 Kavli Nanoscience Institute, California Institute of Technology, 1200 E. California Blvd, Pasadena, CA 91125 USA

**Keywords:** Structural properties, Characterization and analytical techniques

## Abstract

Small organic molecules, like ethane and benzene, are ubiquitous in the atmosphere and surface of Saturn’s largest moon Titan, forming plains, dunes, canyons, and other surface features. Understanding Titan’s dynamic geology and designing future landing missions requires sufficient knowledge of the mechanical characteristics of these solid-state organic minerals, which is currently lacking. To understand the deformation and mechanical properties of a representative solid organic material at space-relevant temperatures, we freeze liquid micro-droplets of benzene to form ~10 μm-tall single-crystalline pyramids and uniaxially compress them in situ. These micromechanical experiments reveal contact pressures decaying from ~2 to ~0.5 GPa after ~1 μm-reduction in pyramid height. The deformation occurs via a series of stochastic (~5-30 nm) displacement bursts, corresponding to densification and stiffening of the compressed material during cyclic loading to progressively higher loads. Molecular dynamics simulations reveal predominantly plastic deformation and densified region formation by the re-orientation and interplanar shear of benzene rings, providing a two-step stiffening mechanism. This work demonstrates the feasibility of in-situ cryogenic nanomechanical characterization of solid organics as a pathway to gain insights into the geophysics of planetary bodies.

## Abstract (web summary)

Solid benzene and other simple organic minerals play important roles in geological processes on Titan-like cold Solar System bodies. Here, the authors discover benzene microcrystals plastically deform via densification enabled by molecule reorientation and shearing.

## Introduction

Simple organic molecules—e.g., ethane^1^ and benzene^2^—exist in gas and liquid phases in Earth’s atmosphere and surface. These molecules are also found to be ubiquitous on another planetary body in the solar system, Titan, the largest moon of Saturn^3^. The Cassini–Huygens mission—two spacecrafts, one for orbiting Saturn, the other for landing on Titan—has revealed that Titan’s thick nitrogen and methane-rich atmosphere enabled dynamic weather, precipitation, and surface liquids. Many eolian, pluvial, and fluvial geological processes, similar to Earth’s, have been observed on Titan to produce rivers, canyons, lakes, and dunes^4^. At the average temperature of ~94 K, Titan’s “hydrology” and landforms consist not of liquid water and silicates, but predominantly liquid methane and a mixture of water ice and solid organics that originated through complex photochemical processes in the upper atmosphere^5^.

Equipped with instruments including the radio detection and ranging instrument (RADAR) and visual and infrared mapping spectrometer (VIMS), Cassini–Huygens mapped the surface morphology of Titan, whose hazy organic atmosphere hinders visual-based observation of the surface. Titan’s surface was identified as being composed of multiple components, including 14% of hummocky/mountainous terrains, which are the oldest on Titan, and 17% of dunes which are relatively young^6–8^. Infrared spectral analysis^9^ and microwave radiometry^10^ reveal the dunes to be comprised of organic materials. Limited data has led to the formulation of multiple competing theories to explain the fundamental mineralogy and geology of Titan. From dune sand transport distances^11,12^, eolian^13^ and fluvial^14^ erosion, karst^15^ and yardang^16^ formation, to putative cryovolcanic processes^17^, many distinguishing mechanisms of these processes are dictated by the largely unexplored fundamental mechanical properties of the frozen organic minerals.

Photochemical models have predicted the formation of benzene in Titan atmosphere^18^ and through a chemical transformation from acetylene in dunes^19^. Single-crystalline benzene (as well as its co-crystals with ethane^3^, acetonitrile^20^, and acetylene^21^, etc.) has been proposed as a potential proxy candidate of the Titan surface mineralogy^5^. Various solid benzene derivatives have demonstrated intriguing mechanical properties. For example, tens-of-micron-wide crystalline hexachlorobenzene exhibits ~360° bending with a local radius of curvature comparable to the width while retaining its macroscopic integrity, presumably enabled by anisotropic supramolecular interactions^22^. Existing studies on solid benzene include high-pressure solid phases of benzene^23–25^, high-pressure high-temperature equation of state calculations^26^ and phase diagram maps^27^, superconductivity of solid benzene under high pressure^28^, stress-induced amorphization^29^, and polymerization of benzene into carbon nanothreads^30^. However, the basic mechanical properties and deformation mechanism of benzene (and of other molecular proxies) in the solid state and at space-relevant temperatures remain open questions and hinder further understanding of the geophysics and surface topographies of Titan and other cold Solar System bodies.

In this work, we freeze liquid micro-droplets of benzene into micro-pyramids, perform uniaxial compression in situ, and corroborate with molecular dynamics simulations, unveiling that crystalline benzene plastically deforms via benzene rings densification, collective reorientation, and gliding according to the local shear direction. We demonstrate the significance of in situ cryogenic nanomechanical characterizations of solid organics towards insights into planetary geophysical studies.

## Results

### Mechanical response of benzene microcrystal under in situ compression

**Fig. 1:**
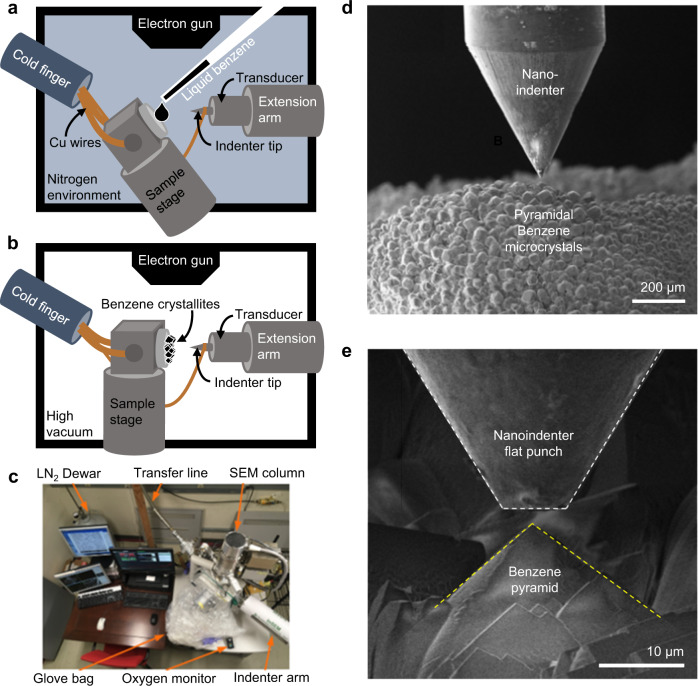
Cryogenic in situ nanomechanical experiment. **a** Schematic of in situ freezing of benzene droplet. **b** Schematic of the cryogenic in situ nanomechanical compression of benzene solid. **c** Picture of the in situ SEM nanomechanics instrument, attached to the glove-bag and connected to the liquid nitrogen cryogenic cooling system. **d**, **e** SEM images of massive pyramidal benzene crystals and one zoom-in benzene pyramid with the nanomechanical indenter flat punch above (the background is shaded, and the indenter and the crystallite are outlined for eye guide).

**Fig. 2:**
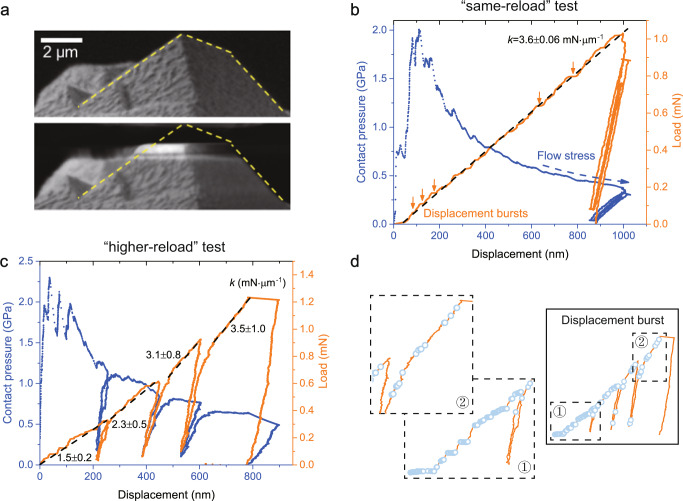
In situ nanomechanical experiment on benzene microcrystals. **a** SEM images of the same microcrystal pre/post-compression (the crystallite is outlined for eye guide). **b**, **c** Load/contact pressure vs. displacement curves of the Same-Reload and Higher-Reload tests. **d** Displacement bursts during a Higher-Reload test.

We performed in situ nanomechanical experiments and atomistic simulations on solid-state benzene microcrystals, as the representative solid organic material. To create solid-state samples, liquid benzene droplets were first dispensed from a pipette onto a standard SEM stub mounted on a liquid nitrogen-cooled cryogenic sample stage in a scanning electron microscope (SEM) chamber within a custom-built nanomechanical instrument connected to a nitrogen-purged glove-bag (Fig. 1a). The chamber was then immediately closed and pumped to ~10^−5^ mbar, while the droplets were flash-frozen on contact with a stage held at ~125 K to form multiple ~10-µm-tall cuboid pyramids (Fig. 1b–e). The sample stage was then tilted to align the micro-pyramids to be amenable to uniaxial compression with initial contact at the apex, deforming uniformly towards the base (Fig. 2a and Supplementary Movie 1). The pyramidal shape of the benzene crystals is practically a nonstandard geometry for mechanical measurements (Supplementary Note 8). It was difficult to reshape or control the geometry as it was formed naturally when the droplets freely fell on the cryogenic stage; still, we need always keep in mind that the plots of posterior force-displacement curves and the way for extracting the modulus are not standard.

Monotonic and cyclical in situ quasi-static micro-compressions on the individual benzene pyramids were performed at a constant loading rate of ~50 µN s^−1^ at ~125–145 K. We prescribed two distinct four-step loading schedules to each sample: (i) loading to a maximum load of ~1.0–1.2 mN, unloading to ~10% of the maximum load, followed by reloading to ~90% the maximum load for three cycles (Same-Reload test, Fig. 2b and Supplementary Fig. 1) and (ii) loading to an initial load ~25% the same maximum load, unloading, and reloading to ~50, 75, and 100% of the maximum load in the three subsequent cycles (Higher-Reload test, Fig. 2c and Supplementary Fig. 1).

Figure 2a shows that the compressed pyramid was flattened from the apex, consistent with a maximum displacement of ~1 μm, with no observable deformation in its volume below the deformation front. The flattened top layer appears brighter in the SEM secondary-electron contrast, which is consistent with a localized densification-type of deformation observed in open-pore foams^31^. Figure 2b, c contains two representative load/contact pressure versus displacement data of Same-Reload and Higher-Reload tests, where contact pressure is calculated by dividing the applied load by the contact area (Supplementary Note 1). Figure 2b and Supplementary Fig. 2 demonstrate that during the initial compression on pristine benzene in the Same-Reload test, the compressive load increased linearly with displacement, and most of the deformation was permanent, with marginal elastic recovery (~15%) after unloading. The deformation behavior in the three subsequent cycles were consistent, exhibiting a common stiffness during reloading and unloading, which suggests that the sequent deformation within the historical maximum was highly repeatable and predominantly elastic in both loading and unloading directions. The contact pressure peaked at ~2 GPa after establishing incipient contact and then monotonically decayed to ~0.4–0.6 GPa at ~1000-nm displacement with the continuously increasing contact area of the pyramid.

Another characteristic of the Same-Reload experiments is the presence of ubiquitous, stochastically distributed, 5–30 nm displacement bursts, marked by arrows over the first loading cycle in Fig. 2b. Despite the noises from cryogenic system vibrations, the displacement bursts could be distinguished through multiple thresholds for the displacement increment between adjacent data points collected at 100 Hz (Supplementary Note 3). In contrast, the displacement bursts are absent in the second-fourth reloading cycles in the Same-Reload test, where the deformation was mainly elastic with negligible hysteresis over a ~85% lower displacement than that in the first cycle. These observations indicate that the permanent densification-induced microstructural deformation occurs nearly entirely during the first cycle, with the deformation in subsequent loading cycles restricted to the previously densified region. Stochastic displacement bursts have indeed been widely observed in nanomechanical studies of metal, especially in single-crystalline metallic nano-pillar compression experiments, where displacement bursts correspond to dislocation avalanches^32–35^. Post-mortem observations always show well-resolved slip lines on the pillar surface— as direct evidence of the deformation mechanism of crystallographic slip via dislocation avalanches. Differently, we did not observe any slip lines in the deformed solid benzene pyramids; instead, the pyramids’ top was flattened in a continuous manner, and the side slopes remained mostly unchanged throughout (Fig. 2a and Supplementary Movie 1), suggesting densification that is distinct from the volume-preserving dislocation plasticity in metallic systems.

Our experiments can be viewed as a reversed classical indentation contact problem where a flat, stiff indenter tip is used to compress the compliant and deformable benzene pyramids. In the mechanical model for indentation, a parameter called the reduced modulus, ${E}_{r}$, is used to describe the stiffness contribution from both bodies through

1 $$\frac{1}{{E}_{r}}=\frac{1-{\upsilon }_{1}^{2}}{{E}_{1}}+\frac{1-{\upsilon }_{2}^{2}}{{E}_{2}}$$

where *ν* and *E* represent Poisson’s ratio and Young’s modulus, and “1” and “2” refer to the two bodies. The expression is symmetric with respect to “1” and “2,” suggesting that ${E}_{r}$ is insensitive to which body is more compliant and that classical nanoindentation theories are appliable to our reversed scenario^36^. The elastic indentation mechanics theory predicts a nonlinear load-displacement relation^36^, while the plasticity events (e.g., the successive discrete displacement excursions, indicated by the pop-ins or displacement bursts) interrupt the nonlinear elastic solution and may explain the linear-like load-displacement relation in the Same-Reload test. Similar linear loading curves were widely observed in single-crystalline nanoindentation tests in metals and small molecules^37–41^.

As for the Higher-Reload tests, we adopt the nomenclature from soil mechanics, where the preconsolidation (i.e., the historical maximum) stress dictates the transition from preceding (elastic) to virgin (plastic) consolidation (i.e., removal of pore volume)^42^. The benzene pyramids experienced such tangent stiffness enhancement during each Higher-Reload reloading cycle (Fig. 2b, Supplementary Note 2, and Supplementary Figs. 3, 4) that the elastic compression from each previous cycle transitions to the onset of plasticity with further loading, and compression then progress into the deeper, undeformed layers of benzene. This transition is also evident in the scarcity of displacement bursts during preceding elastic compression and their emergence during further virgin compression (Fig. 2d and Supplementary Fig. 5). We observed systematic stiffening in each consecutive loading of the virgin material during Higher-Reload cycles, with the average stiffness progressively increasing by half, one-third, and one-eighth of the previous value, respectively. The increasing stiffness per cycle—indicating overall nonlinear load-displacement relation—is consistent with the elastic response of the densified region from preceding loading cycles. A similar stiffening phenomenon was observed during nanoindentation of hexachlorobenzene crystals^22^.

### Crystallinity of the cryogenically formed solid-state benzene

**Fig. 3:**
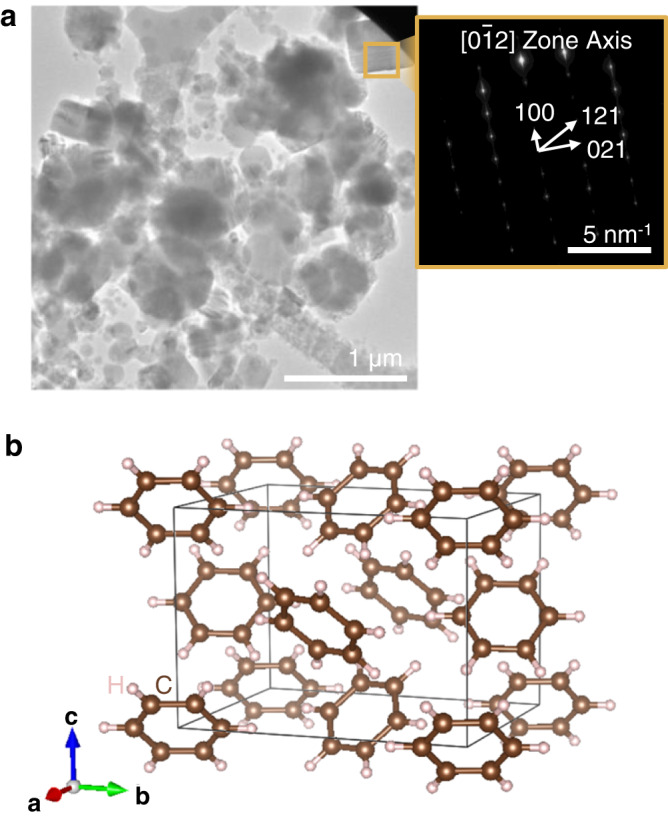
Microstructural and phase characterization. **a** Cryo-TEM bright-field (BF) image of tens of benzene crystallites; inset: SAD determining the crystal structure. **b** The atomic model of one unit cell of Pbca-phase solid benzene.

The microstructure of solid benzene was characterized using cryo-transmission electron microscopy (cryo-TEM) on similar droplets solidified directly on the lacey carbon TEM grid. Due to the lower volume of liquid benzene needed in comparison to the in situ SEM sample preparation, the TEM sample contained smaller solid benzene particles. Nevertheless, the SEM and TEM samples shared comparable cryogenic temperature profiles, which is important in guaranteeing consistency in phase formation. Selected area diffraction (SAD) was performed for a single grain solidified on the TEM grid (Fig. 3a). Using the lattice parameters of benzene, *a* = 7.384 Å, *b* = 9.416 Å, and *c* = 6.757 Å^43^, the diffraction spots (Fig. 3a, inset) were indexed as (100), (021), and (121) with >97.5% match for the [0$\bar{1}$2] zone axis. The findings suggest that the benzene pyramids are composed of single crystals, with the orthorhombic Pbca structure, identified as the only equilibrium crystalline phase of benzene under present SEM and TEM conditions^23^, i.e., ~77–145 K in vacuum (Fig. 3b).

### Atomistic densification mechanism of solid-state benzene

**Fig. 4:**
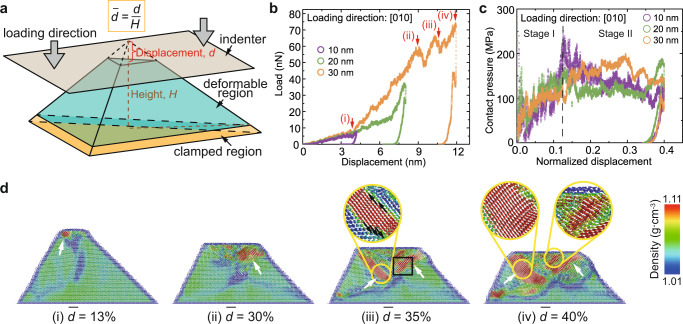
MD simulations on pyramidal benzene. **a** Schematic of the simulation on pyramidal benzene. **b** Load vs. displacement and **c** contact pressure vs. normalized displacement curves of the simulations on pyramidal benzene samples with the loading direction of [010] and the height *H* = 10, 20, and 30 nm. **d** Sequential snapshots of the density distribution of a cross-sectional slice cut from 30-nm-height pyramidal benzene; insets in (iii) and (iv): reorientation-induced densification.

We employed large-scale molecular dynamics (MD) simulations to investigate the molecular-level origin and mechanism of the solid benzene’s plastic deformation and stiffening behaviors^44^. The simulated samples consisted of periodically stacked Pbca-phase benzene crystals, consistent with the experimental characterization. Figure 4a shows the deformable region of the simulated pyramidal sample, with a height *H*, which resides on a fully clamped bottom layer. Figure 4b contains the load vs. displacement response of three samples with different heights, *H* = 10, 20, and 30 nm under fixed [010] loading direction and 10 K temperature (Supplementary Movies 2–4). This plot demonstrates that the initial load-displacement response, up to a displacement of ~4 nm, is linear-like and approximately identical for all samples, consistent with the experimental observations. All samples exhibit subsequent stiffening, which commences at lower displacements, $d$, as the sample height increases. Figure 4c conveys the contact pressure, calculated from the instantaneous contact area *A*, plotted against the normalized displacement $\bar{d}$, defined as the absolute displacement divided by the overall height, and reveals a roughly two-stage evolution during deformation: (1) a linear increase in contact pressure up to a normalized displacement of 0.12, with a virtually identical response among all samples up to this normalized displacement and (2) a plateau stage, where the contact pressure remains relatively constant at ~150 MPa, with fluctuations that are caused by the sudden appearance of the avalanche of densified benzene molecules (Fig. 4d and Supplementary Movie 4).

Figure 4d presents sequential snapshots of a cross-sectional slice from the sample with a height *H* = 30 nm during compression, colored by the local actual density, of which the calculation method based on the regional atom distributions are described in Supplementary Note 6, illustrating a heterogeneous densification profile. In all simulations, the initial densification region, marked by the arrow in Fig. 4d(i), always nucleated at the apex of the pyramid and expanded roughly layer-by-layer with further compressive load, with a maximum density contour concentrated directly underneath the indenter ((i) in Fig. 4d and Supplementary Movies 2–4). The initial densification occurred at a normalized displacement of 0.12, within the first stage of contact pressure evolution for all sample heights. After the onset of stiffening, the densification region further extended from the edges of the flattened top towards the undeformed layers below, along the 45°-tilt lines (white arrows in (ii)–(iv) of Fig. 4d) and progressively thickened.

**Fig. 5:**
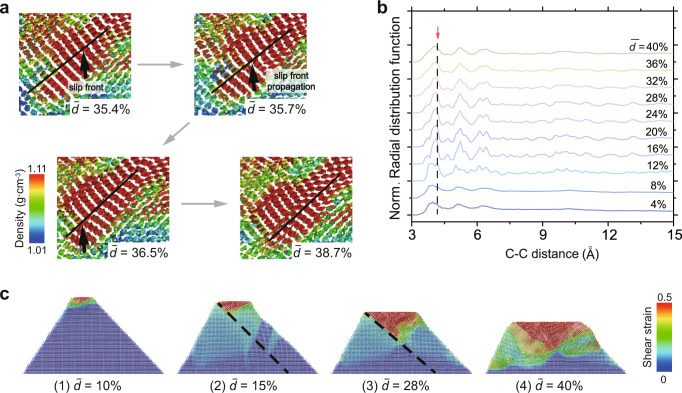
Atomistic deformation mechanism in crystalline benzene. **a** An intermolecular slip process in the black-boxed region in (iii) of Fig. 4d. **b** Radial distribution function of the densified region at increasing normalized displacements in the 30-nm-height sample loading from [010]-direction; the red-arrowed dash line tracks the feature peak. **c** The evolution of the shear-strain distribution of the sliced sample.

The mechanism of densified region formation appears to occur in two steps—through firstly, collective reorientation of benzene rings along the 45° local shear direction (Fig. 4d (iii), circled region) and secondly, interplanar shear slipping between paralleled molecules (Fig. 5a and Supplementary Movie 5). The reorientation front (black arrows in Fig. 4d(iii), circled region) propagated primarily along the 45°-direction to extend the rows of coplanar benzene rings before deepening into the next parallel crystallographic plane (Supplementary Fig. 6). Two obvious, large load drops are marked with (ii) and (iii) in Fig. 4b and are related to the sudden reorientation-induced densification and propagation of the densified phase (Fig. 4d and Supplementary Fig. 6). At the normalized displacements of $\bar{d}$~35.4-36.5%, the interplanar shear front field (arrows in Fig. 5a) propagated analogously to dislocation in atomic crystals, driving offsets above and below the plane of shear in every column of benzene rings that it passed. At relative displacements greater than $\bar{d}$~36.5% when the offset almost equated to half of the column spacing, the face-to-face π–π interactions between the stacked benzene rings^45^ likely caused the front to expand into a slip band, which distributed the shear offset over multiple neighboring layers, relieving the high local strain and accommodating the increasing global strain while preserving the original ring-to-ring connectivity. The radial distribution function (RDF) analyses of the densified region is consistent with this mechanism (Supplementary Note 7 and Fig. 5b; the selection of densified region is referred to in Supplementary Fig. 7). During compression, the diffuse RDF peaks became sharp at $\bar{d}$ ~8–12%, matching the onset of contact pressure plateau. The RDF peak shapes and positions remained stable until $\bar{d}$ ~32–36%, suggesting stable propagation of the densification region. The highest RDF peak at a carbon-carbon pair distance of ~4.1 Å, which also experienced the most significant intensity increase, likely correlated to the interplanar spacing after the benzene rings’ reorientation. Beyond $\bar{d}$ ~32–36%, the RDF peaks became diffuse again, which could be explained by interplanar shear slipping and distorting the columns of stacked benzene rings (Fig. 5a).

The snapshots of the shear-strain evolution of the same slice, shown in Fig. 5c and Supplementary Movie 5, revealed that the high shear-strain region initiated from the apex and then propagated along an inverted triangular shape where the benzene molecules assumed a largely distorted arrangement— at $\bar{d}$ ~10%, forming a 45°-shear band which guided the region above to slip along and plateaued at a contact pressure of 150 MPa ((2) to (3) in Fig. 5c). The reorientation-induced densification region appears to be located directly below the shear band, which suggests that the role of the shear band is to facilitate the reorientation of the benzene molecules and to thicken the densification region (comparing (iv) in Fig. 4d and (4) in Fig. 5c). Distinctively, the interplanar slip/distortion in the densification region induced large shear strain evident in the contour maps.

The formation of shear bands oriented at 45° with respect to the loading direction is caused by the motion of loosely stacked Van der Waals force-bonded benzene rings along the directions of maximum shear-compoenent that caused ring reorientation. This phenomenon is distinct from the shear slip in single-crystalline metals, where dislocation glide on specific crystallographic slip systems defines plastic deformation, with minimal plane/lattice rotation, as evidenced by a similar compression experiment on single-crystalline gold pyramids^46^. The gold pyramid study reported conserved sample volume before and after compression, consistent with the dislocation glide mechanism; in contrast, the aromatic rings in solid benzene were loosely stacked and bonded by weaker Van der Waals forces, which enables densification via ring re-arrangements.

The formation of the densified region and shear band in MD simulations could explain the predominantly plastic deformation in experimental observation; meanwhile, the densified regions induced by reorientation and interplanar slip contribute to the stiffening behavior in the load-displacement curves. The molecular-level deformation behavior—reorientation and interplanar shear of benzene rings with respect to the local stress field—is likely generalizable to different sample geometry and loading conditions. In simulations, varying the unit cell orientation inside the pyramid (Supplementary Fig. 8) or the cryogenic temperature in the range of 10–30 K barely affected the deformation behavior due to the highly responsive reorientation of benzene molecules under the applied stresses (Supplementary Fig. 9).

Our in situ SEM observations reveal the entire deformed pyramid, including the densified region at the top, to be thermodynamically stable after unloading because we did not detect any shape change. RDF evolution of high shear-strain region with shear strain larger than 0.5 from MD simulations in Supplementary Fig. 11 reveals no obvious peaks, which is consistent with amorphous-like microstructure. To be noticed, The uniaxial pressures in our experiments (*P*_max_ = 2.0 GPa) and simulations (*P*_max_ = 0.2 GPa) were 8% (based on experiments) as those needed to trigger amorphization (*P*_amorphization_ ~25 GPa) and 20% below the pressure to initiate phase change from Pbca to P2_1_/*c*^23,29^. This renders the discussion on whether amorphization or a phase change occurred in our experiments inconclusive; instead, we describe the microstructure as amorphous-like.

## Discussion

We have demonstrated the feasibility of nanomechanical experiments on individual solid benzene micro-crystallites and the atomistic origin of the observed deformation behavior. The data provide insights into the mechanical properties of other simple-organic materials representing the surface mineralogy of cold Solar System bodies like Titan. These materials are characterized by strong intermolecular forces, e.g., acetonitrile with two-dimensional hydrogen-bond networks^47^, potentially coordinating the collective molecular responses in an analogous way as the π–π stacking in solid-state benzene dictates the reorientation and shearing. In large-organic-molecule crystals, however, significant molecule rotation is typically hindered by steric constraints, with the deformation mechanisms dependent on the pre-existing interatomic forces in the original crystal structure: hexachlorobenzene, with column-stacked rings, is highly resilient to plastic bending^22^; 1,3,5-trichloro-2,4,6-triiodobenzene, with strong 2D networks, and crystals with intralayer/interlayer stacking sequences experience plastic shearing in-plane or along weak interlayers^48^. On the other hand, the proton-disordered phase-I_h_ ice microfiber with a diameter of less than 10 microns, as a small-molecule inorganic crystal, can sustain an elastic limit of up to 10.9% under bending deformation along with a pressure-induced phase transition from I_h_ to II^49^. MD simulations of phase-I_h_ ice demonstrated that under compression, the column of oxygen atoms parallel to the compression direction would re-orient into a regular zigzag pattern^50^, and that under nanoindentation near the melting temperature, a quasi-liquid layer was formed at the ice-indenter interface by pressure-induced amorphization^51^. These previous studies suggest the clear difference between the deformation of small- versus large-molecule crystals and the special significance of intermolecular interactions in the nanomechanical response of small-molecule crystals, organic or inorganic. Furthermore, the group of noble-gas solids, like hexagonal-close-packed (hcp) and body-centered cubic (bcc) ^3^He, hcp ^4^He, face-centered cubic (fcc) Ne and Ar etc., can only keep in solid phase with crystalline structure at low temperatures or high pressures, the same as benzene molecules^52,53^. By some mechanical experiments like pulling a ball through the solid hcp ^4^He^54^, pulling a superconductive wire through bcc ^4^He^55^, or uniformly shearing hcp ^4^He triggered by piezoelectric transducers^56^, the solid helium always mechanically responded in plastic flow, phenomenologically similar to solid benzene in our experimental testing. Due to the metal-like atomic configuration, the plastic deformation can be explained by the avalanches of creation, multiplication, and interaction of dislocations in solid He. However, although the tested micro-crystallite benzene was also arranged in an orthorhombic structure, the different orientations of the benzene molecules in one lattice and the tendency towards π–π stacking between benzene rings made it plastically deform in different underlying mechanisms compared to noble-gas solids, in which every atom is identical with no orientational characteristics.

Despite the nonstandard geometry of the micro-crystallite for extracting the modulus, hardness, etc., a longitudinal modulus of ~0.7–1.8 GPa is estimated for the densified region of solid-state benzene, by applying several approximations (Supplementary Note 4 and Supplementary Fig. 10). The upper-bound value is ~55–70% lower than Young’s modulus in the lattice-normal directions calculated from the elastic tensor measurement by an ultrasonic pulse method^57,58^ (data summarized in Supplementary Table 1). This inconsistency in the modulus may be due, in part, to the differences in sample size, microstructures, crystallographic orientations, porosity, and fabrication methods used in the described measurement techniques. This range of the modulus is generally comparable to the nanoindentation-measured Young’s modulus of coronene (a polycyclic aromatic hydrocarbon) and biphenyl (two benzene molecules linked by a single bond). It is an order of magnitude lower than that of water ice at 94 K, laboratory Tholin (photochemically-formed disordered polymer-like organics), and white gypsum sand, and two orders of magnitude lower than that of basalt and silica sand^12^. While the modulus was relatively low, the solid benzene pyramids demonstrated substantial densification without fracture as flow stresses asymptotically approached a high value of ~0.4–0.6 GPa. At the microscale, the shear band formation facilitating reorientation and releasing local stress concentration might imply good resilience to fracture of small benzene micro-crystallites and viability of particle compaction and transport on the cold planet surface. Additionally, densification could occur under the planet’s surface if benzene crystals were subjected to pressures during impact or burial^59^ and/or subjected to higher pressures; the non-recoverability after pressure removal, as shown in the experiment, would be of interest if materials at depth were eventually exposed (via geologic uplift^60^) onto the surface.

With further studies and an understanding of the plastic size effects, the present findings could help the development of continuum and discrete-element-method (DEM) models to simulate planetary geological processes, including the deposition, compaction, and lithification of solid organic materials and spacecraft landing missions on cold Solar System bodies. While this study focused on small-scale testing and simulations of solid-state benzene as a starting point, future studies could explore (i) the plastic size effects in these materials; (ii) the fracture toughness of solid-state benzene to further infer its robustness in more violent geological processes; and (iii) the fundamental mechanical properties of myriad small-molecule organic solids.

Through revealing the deformation characteristics of solid-state benzene at the smallest scale, we suggest (i) the critical role of intermolecular forces in dictating a two-step (reorientation and shear) deformation mechanism of this representative small-molecule single-crystal, in comparison to the dislocation-mediated plasticity in atomic single-crystals consisting of nondirectional atoms and (ii) the potentially unique behaviors of the exotic simple-organic minerals compared to hard and stiff siliceous rocks of Earth. We anticipate that these implications will encourage future studies to advance the fundamental mechanics of small-molecule crystals and unveil the mysteries behind planetary geological processes.

## Methods

### In situ nanomechanical experiments

The compression experiments were performed with an in situ nanomechanical instrument^61^, which (i) enables applying mechanical loads with a prescribed schedule, (ii) allows simultaneously collecting mechanical data and observing the deformation in SEM, and (iii) functions at a wide temperature range. The schematic of the experimental setups is shown in Fig. 1 in the main text.

During the experiment, the sample holder (Ted Pella standard pin mount aluminum SEM stub) was cooled to ~125 K with a copper cooling line (40 to 400 K capability from Janis Research Company) chilled by liquid nitrogen in an SEM chamber (Quanta 200, Thermo Fischer Scientific). The chamber is evacuated (~10^−5^ mbar) and equipped with a nanomechanical testing module (InSEM^TM^ from Nanomechanics Inc.; force noise floor <200 nN; displacement noise floor <0.1 nm). After pre-cooling the sample holder, the chamber was then opened into an N_2_-purged glove-bag (oxygen levels below 2% measured by an MSA ALTAIR® O_2_ Pro) to minimize water condensation onto the cooled components. Several milliliters of anhydrous benzene (Sigma Aldrich 99.8%) were dropped onto the sample holder tilted by ~30° relative to the floor. Until a visibly sufficient amount of benzene was frozen on the sample holder, the chamber was closed and re-evacuated. The stage temperature typically increased from ~125 to ~180 K during the short window when the chamber door was opened to freeze the sample and then decreased after the chamber was closed and re-equilibrated for ~2 h.

The sample was scanned with SEM to identify crystals whose cuboid-corner closely points towards the indenter; then, the corner orientation was manually adjusted to minimize possible misalignment. Compressions of individual cuboid solid benzene crystals were performed with a ~7-µm-diameter diamond flat-punch indenter tip. Two cyclic loading profiles were employed: repeated compressions to a particular load (referred to as Same-Reload tests) and successive compressions to progressively higher loads (referred to as Higher-Reload tests) (Supplementary Fig. 1). The same-reload experiments were conducted at temperatures of 123.39 ± 0.99 K, and the higher-reload ones at 144.40 ± 0.35 K (Supplementary Note 5). The constant loading rates were 41 or 61 µN s^−1^ up to maximal loads of ≤1.5 mN. The data collection rate was 100 Hz. The results are summarized in Supplementary Figs. 2,3. Note that the noisiness in the raw data primarily resulted from the liquid N_2_ influx and evaporation in the cryostat installed on one of the ports in the SEM chamber.

### Microstructure analysis via cryo-TEM

An additional solid benzene sample was prepared for microstructural analysis via cryo-TEM by first blotting 3 µL of liquid benzene on a lacey carbon TEM grid at a blot force setting of −15 at 10 °C for 0.5 s in an FEI Vitrobot Mark IV and then plunging it into liquid nitrogen. The sample was kept in liquid nitrogen during transfer to the TEM (Talos Arctica, Thermo Fisher Scientific) and imaged at 77 K with the field emission gun operating at 200 kV and a 4k × 4k FEI Falcon II direct electron detector. A 10-µm selected area diffraction (SAD) aperture was used to isolate information from an individual crystal during diffraction pattern acquisition.

### Atomistic simulations

We carried out a series of large-scale atomistic simulations to emulate the uniaxial compression of solid benzene corners using LAMMPS^44^. In all simulations, a reactive potential for hydrocarbons with intermolecular interactions, also called AIREBO potential^62^ was used to describe both the intramolecular and intermolecular interactions. Such force field can both describe the covalent bonding interactions and the torsion, dispersion, and nonbonded repulsion interactions via an adaptive treatment, which enables it to capture the chemical reactions, e.g. the breakage and formation of the carbon-carbon bonds, in the hydrocarbon system. The corner sample was extracted by cutting a triangular pyramid shape from bulk solid benzene with the crystalline phase Pbca (lattice parameters of 7.644, 9.514, and 6.720 Å), approximately consistent with the phase characterization in SAD (Fig. 3a). The heights of the corner samples were determined as 10, 20, and 30 nm separately, further to perform uniaxial deformation simulations. We selected three loading directions: [010], [110], and [011], which was perpendicular to the base of the triangular pyramid. The orientation of lateral faces was shown in Supplementary Fig. 8. At the bottom of the sample, the section of one-tenth height was fully fixed; the upper section was deformable. The corner sample was relaxed at 10 K for 200 ps under a canonical (NVT) ensemble. During the deformation process, a rigid indenter was used to compress and release the corner sample at a loading rate of 5 × 10^8^ s^−1^ up to 40% of the total height. The temperature effect on the deformation process was also investigated. The temperature during the simulations was fixed at 10, 20, and 30 K separately to repeat all the simulations with samples of different heights and different loading directions described above. During the simulations, the force exerted on the indenter in the loading direction was outputted as the external force exerted on the overall sample so that the load-displacement curve for the simulation can be obtained.

Subsequently, we used OVITO software to post-process the simulation results^63^. We transferred the load-displacement curves to the contact pressure-normalized displacement curves for convenient comparisons between simulation cases on different sample sizes. The contact area was determined by making a surface mesh from the full atomic models with the OVITO package. The contact pressure was calculated as the load divided by the contact area; the normalized displacement was calculated as the displacement divided by the initial height of the triangular pyramid. The shear strain were also calculated in OVITO for further coloring the atoms^64^.

## Supplementary information

Supplementary Information

Description of Additional Supplementary Files

Supplementary Movie 1

Supplementary Movie 2

Supplementary Movie 3

Supplementary Movie 4

Supplementary Movie 5

## Data Availability

The processed experimental data and simulation videos are provided in the Supplementary Information. The raw data are protected and are not available due to data privacy restrictions. Correspondence and requests for materials should be addressed to W.Z.
