# Supplementary material for: Deformation characteristics of solid-state benzene as a step towards understanding planetary geology
Source: Nat Commun. 2022 Dec 26;13:7949. doi: 10.1038/s41467-022-35647-x (PMC9792550; doi:10.1038/s41467-022-35647-x)
Supplement: Supplementary file 1 — Supplementary Information [file 41467_2022_35647_MOESM1_ESM.pdf]

Supplementary Information for

Deformation Characteristics of Solid-state Benzene as a Step towards Understanding Planetary Geology

Authors: Wenxin Zhang[1]†*, Xuan Zhang[2]†, Bryce W. Edwards[1], Lei Zhong[3], Huajian Gao[4,5,3], Michael J. Malaska[6], Robert Hodyss[6], Julia R. Greer[1,7]

Affiliations:

[1]Division of Engineering and Applied Sciences, California Institute of Technology, 1200 E. California Blvd., Pasadena, CA 91125, USA.

[2]INM—Leibniz Institute for New Materials, Campus D2 2, 66123 Saarbrücken, Germany.

[3]School of Engineering, Brown University, Providence, Rhode Island 02912, USA.

[4]School of Mechanical and Aerospace Engineering, College of Engineering, Nanyang Technological University, 70 Nanyang Drive, 639798, Singapore.

[5]Institute of High Performance Computing, A*STAR, 138632, Singapore.

[6]Jet Propulsion Laboratory, California Institute of Technology, 4800 Oak Grove Drive, Pasadena, California 91109, USA.

[7]Kavli Nanoscience Institute, California Institute of Technology, 1200 E. California Blvd., Pasadena, CA 91125, USA.

*Corresponding author. Email: wzhang2@caltech.edu

†These authors contributed equally to this work.

This file includes:

Supplementary Notes 1 to 8
Supplementary Table 1
Supplementary Figures 1 to 11

Supplementary Note 1. Calculation of Contact Pressure in Experiments. The pyramidal sample geometry lends itself to the calculation of contact pressure, as representation of the compressive stress. Contact pressure $P_{contact}$ is defined as the ratio between the real-time load F and the instantaneous contact area $A_{contact}$ between the indenter tip and the sample, or

$$P_{contact} = F/A_{contact} \qquad (1).$$

For an ideal cuboid-corner pyramidal geometry, at displacement h,

$$A_{contact} = 3^{3/2}h^2/2 \qquad (2).$$

Given minimal bulging was observed in SEM during compression, the self-similar cross-section assumption is valid. For the real experimental data, the finite curvature at the apex of the crystal is taken into account. The adjusted contact area is

$$A_{contact} = 3^{3/2}(h+\delta)^2/2 \qquad (3),$$

With $\delta < 50$ nm based on specific SEM observation. The contact pressure is then

$$P_{contact} = 2F/[3^{3/2}(h+\delta)^2] \qquad (4).$$

Supplementary Note 2. Calculation of Loading Stiffness for Virgin-compression in Higher-reload Experiments. For the first loading cycles, due to the linearity of the loading segment, the stiffness was taken as the best-fitted slope of the line. Whereas, for the reloading cycles, the virgin-compression is considered as the loading segment from where the current load just surpassed the historical maximum to unloading. And the loading stiffness is then found as the best-fitted slope of this segment. Note that the displacement from the bursts is included in calculating the loading stiffnesses, which could cause these values to slightly underestimate the stiffness of the pre-burst pristine benzene. The results are shown in Supplementary Fig. 4.

Supplementary Note 3. Identification of Displacement Bursts for the Loading Segments in Higher-reload Experiments. Based on the current loading rate, data acquisition frequency, and cryogenic system's vibration magnitude, the criteria for displacement burst identification are: (to simplify notation, total cumulative displacement is called *h*, and the incremental displacement per data point is called Δ)

(a) $\Delta > \delta = 0.7$ nm, i.e., approximately twice median Δ (median instead of mean is considered because median is less sensitive to cryogenic vibrations) as well as the unit cell size of benzene;

(b) the mean Δ from the adjacent five data points should be greater than $\delta/3$;

(c) for all data points identified by criteria a & b, the last identified point should be separated by no more than one data point from the current identified point;

(d) *h* at the current data point identified by criteria a, b, & c should be greater than *h* at the last data point identified by criterion a, b, & c;

(e) the mean *h* of the adjacent seven data points is less than the mean *h* of the next seven data points.

The criteria b-e partially serves for removing false idenfication caused by vibration. Still, very occasionally the criteria falsely identified artificial displacement bursts or omits likely real displacement bursts satisfying criteria a & b, in which case the relevant data points were visually examined and manually removed/reselected. The results are shown in Supplementary Fig. 5.

Supplementary Note 4. Estimation of the Longitudinal Modulus of the Densified Region.

We estimated the longitudinal modulus of the densified region along the axial compression direction from the unloading segment of the load-displacement data and the post-mortem SEM images:

$$E_l = kH(1 - D_{pl}/H)/A = ckH/2.6h \qquad (5),$$

where D_{pl} is the plastic displacement, H is total deformed height, k is the unloading stiffness, A is the cross-sectional area calculated at distance h from the initial apex location, and

$$c = 1 - D_{pl}/H \qquad (6)$$

represents the densification ratio. Assuming linear elastic unloading and approximating cross-sectional area of the compressed pyramid as a constant (in reality, it had a slight variation over the height due to the small taper of the side), E_l is estimated as ~0.7-1.8 GPa for $c \sim 0.4 \pm 0.04$ (Supplementary Fig. 10).

Supplementary Note 5. Influence of Temperature on the Mechanical Properties. As applied in a previous room-temperature nanoindentation study of a selection of candidate Titan minerals[12], the Young's modulus E scales as a function of temperature T as

$$E(T) \cong E_0(1 - T/2T_m) \qquad (7),$$

where E_0 is the Young's modulus at 0 K, and T_m is the melting temperature. Applying this relation to the benzene crystallites in this work, whose melting temperature is 278.7 K [27], we find that E(94 K) ≅ 1.07 E(123.39 K) ≅ 1.12 E(144.40 K). This implies that the Young's modulus at Titan's surface temperature is only ~12% below the one measured in our work. The agreement should hold for the longitudinal modulus, as well because it is linearly related to the Young's modulus through a geometric factor independent of temperature. Yu et al. also suggested the empirical relation between hardness H and Young's modulus E through a power-law scaling, with a close-to-unity power coefficient[12], further suggesting that the hardness as well as the strength have similarly weak temperature-dependence and validates our measurements.

Supplementary Note 6. Calculation of Density Distributions from MD results. We used the following method to plot the density distribution: we calculated the coordination number (CN) of Carbon atoms around one Carbon atom within a cut-off of 8 Å and calculate the density of this position by the equation:

$$\rho_{Atom} = \frac{(\mathrm{CN}+1)\times 13\times m}{4/3\times\pi R^3} \qquad (8)$$

Where *m* is the atomic mass constant, which is 1.66×10^{-27}kg, *R* is the cut-off radius. By substituting the initial lattice constants of the solid benzene for MD simulations, we can calculate the initial density of the sample as:

$$\rho_{initial} = \frac{24\times 13\times m}{abc} = 1.05977\ \mathrm{g\ cm^{-3}} \qquad (9)$$

which is consistent with the initial density distribution in MD videos (Supplementary Movies 2 to 4).

Supplementary Note 7. Calculation of Radial Distribution Functions from MD results.

Based on observations, we isolated the molecules of the densification regions by selecting the atoms in OVITO that satify non-affine square displacement >100 and shear strain <0.5 with cuf-off radius of 10 Å (see Supplementary Fig. 7 for example). And then the RDF of C-C pairs was calculated in OVITO, setting the cut-off of 15 Å (Fig. 5b and Supplementary Fig. 11). All curves are normalized by the height of the first peak.

Supplementary Note 8. Complexity of stress distribution due to “pyramid”-shaped solid benzene. The stress tensor within the pyramid is non-uniform when subjected to a uni-directionally applied external load because of the increasing cross-sectional area during compression. The pressure distribtuion in the loading direction decreases with the rising cross-sectional area, with the largest local deformation occurring at the maximum-pressure region, i.e., the apex. The non-negligible shear stress component perpendicular to the laoding direction arises as a result of transverse expansion of adjacent atomic planes under pressure.

Supplementary Table 1. Elastic constants of solid benzene[57].

	c_{11}	c_{22}	c_{33}	c_{44}	c_{55}	c_{66}	c_{12}	c_{13}	c_{23}
170 K	8.01	9.26	7.88	3.18	5.53	1.95	3.85	4.80	5.08
138 K	8.61	10.01	8.63	3.56	6.13	2.10	4.15	5.10	5.38

Units: GPa

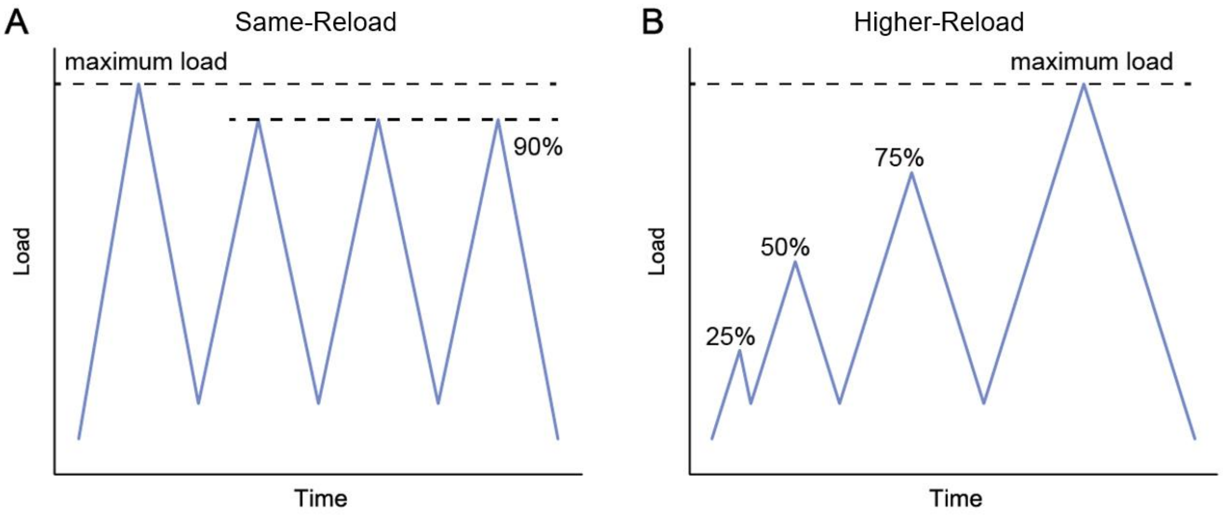

Supplementary Figure 1. The loading profiles for (**A**) the Same-Reload test and (**B**) the Higher-Reload test.

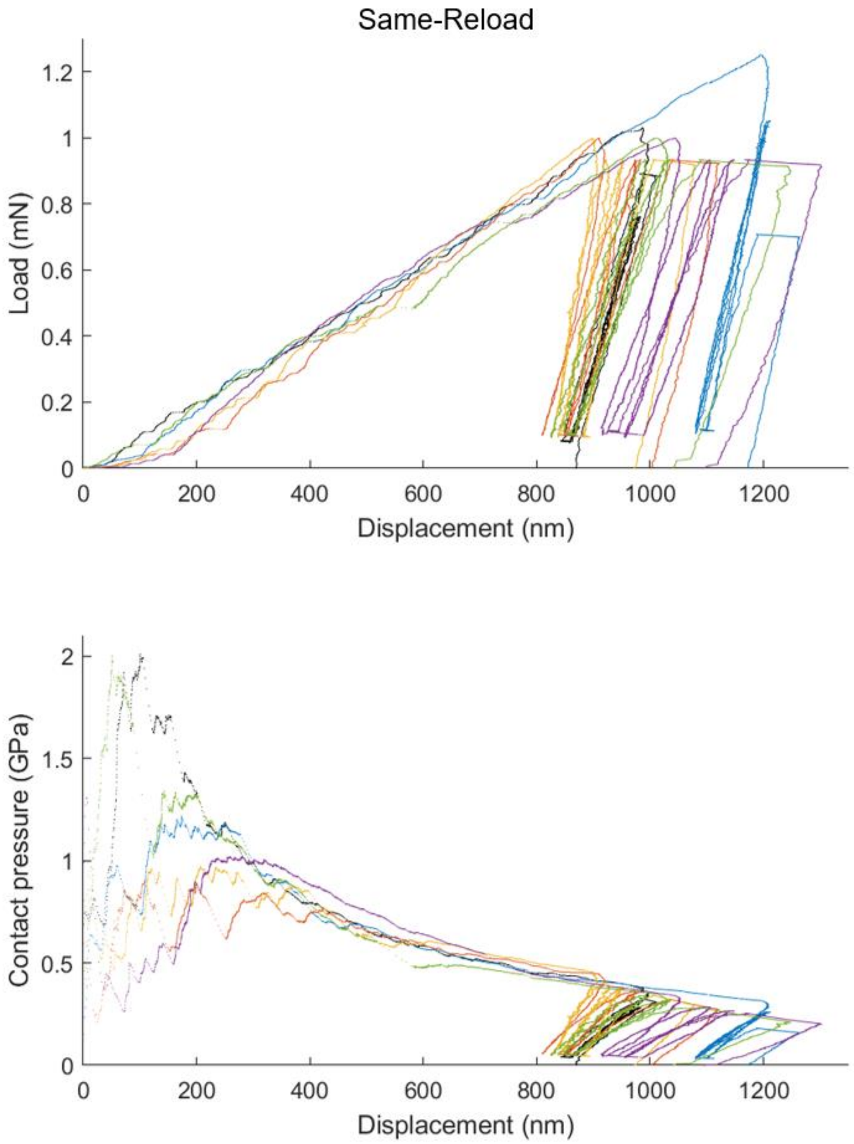

Supplementary Figure 2. Same-Reload tests. Load-displacement raw data and calcualted contact pressures.

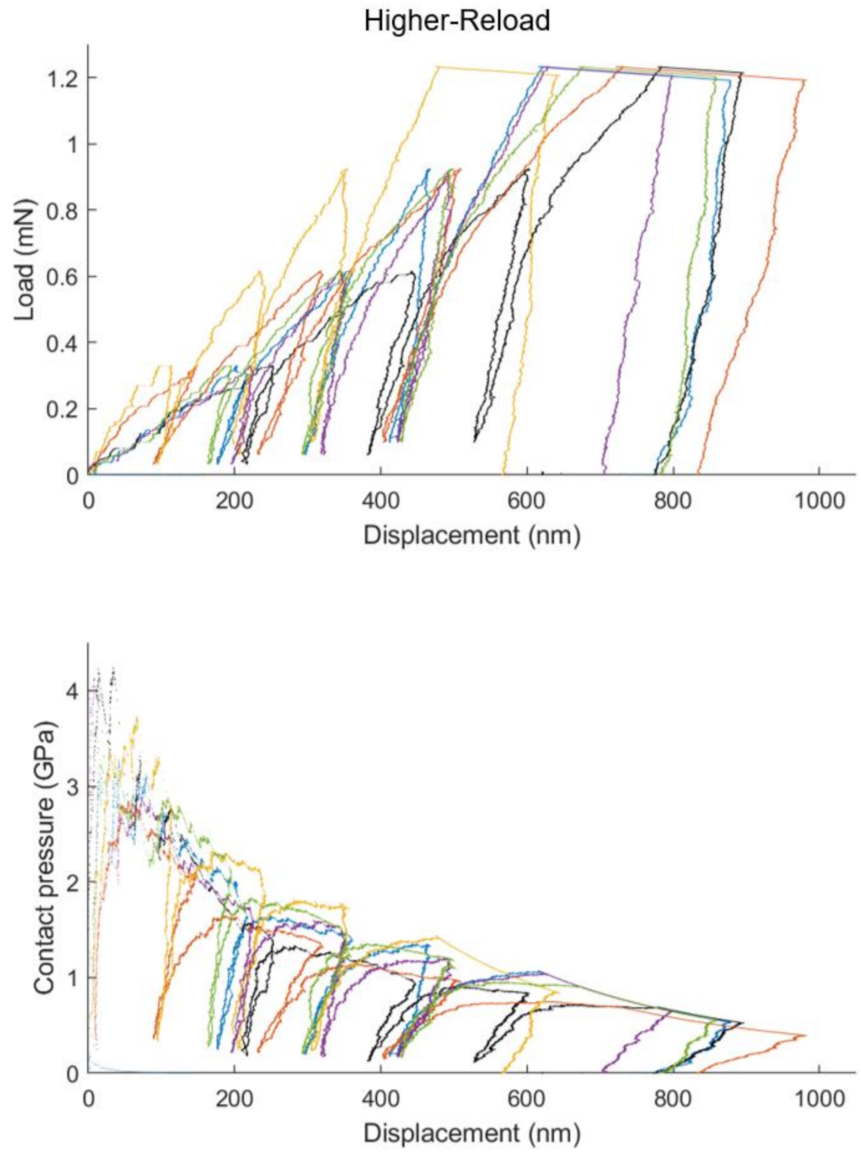

Supplementary Figure 3. Higher-Reload tests. Load-displacement raw data and calcualted contact pressures.

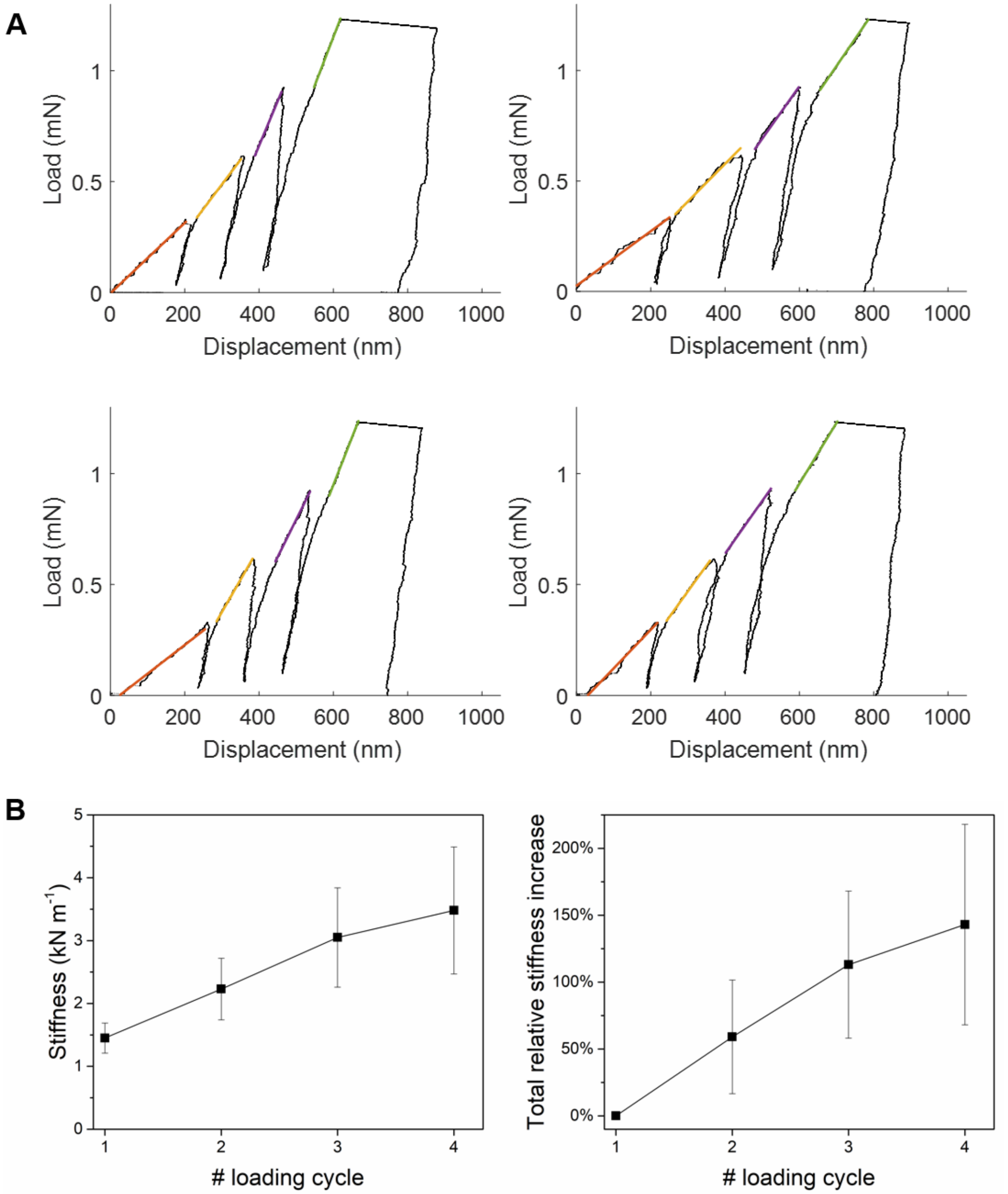

Supplementary Figure 4. Higher-Reload virgin-compression stiffness (outliers removed): (**A**) fitting results for individual datasets; (**B**) comparison of the stiffening effect over loading cycles. Error bars represent the standard deviation of each measurement.

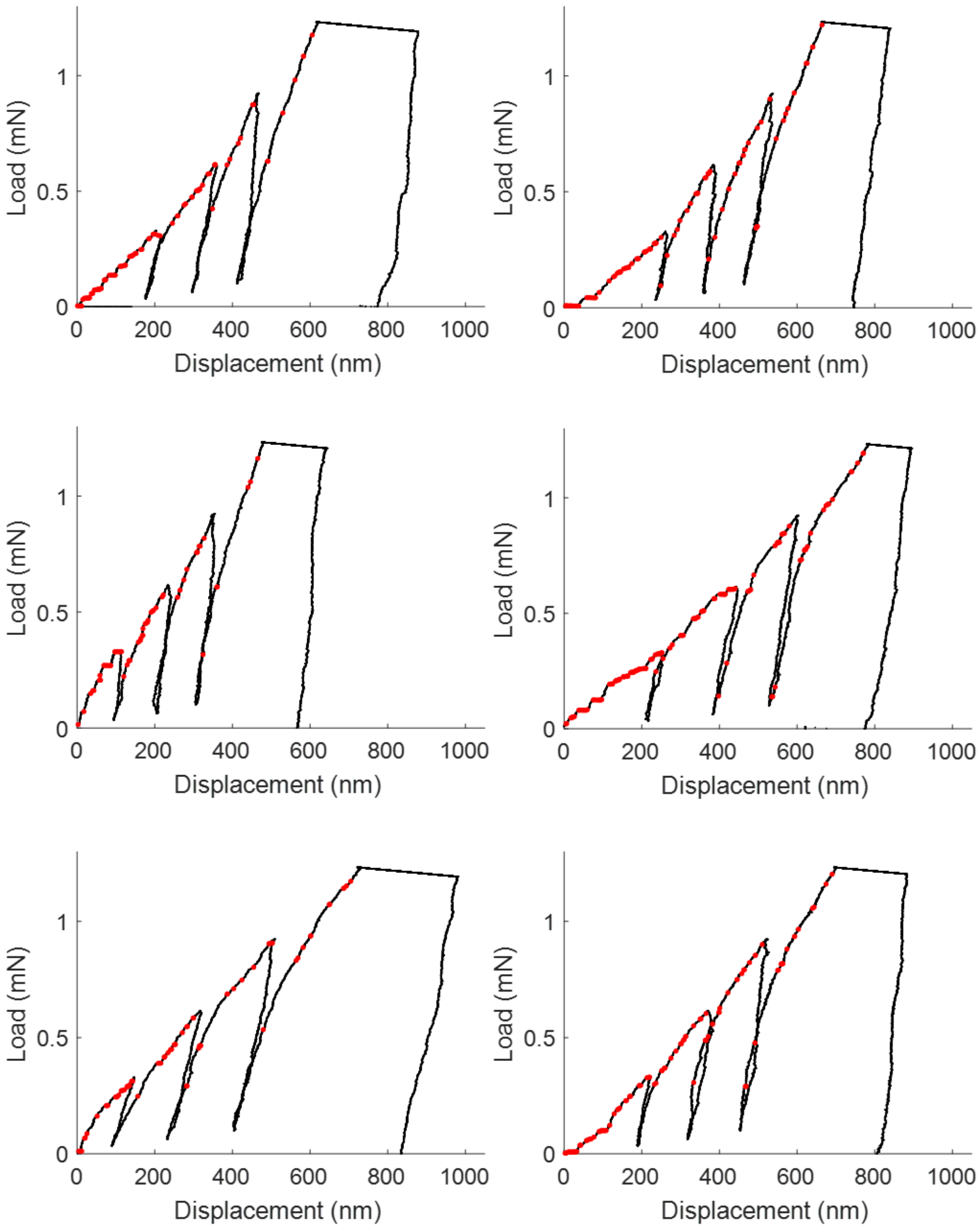

Supplementary Figure 5. Identification of displacement bursts (red) for Higher-Reload experiments.

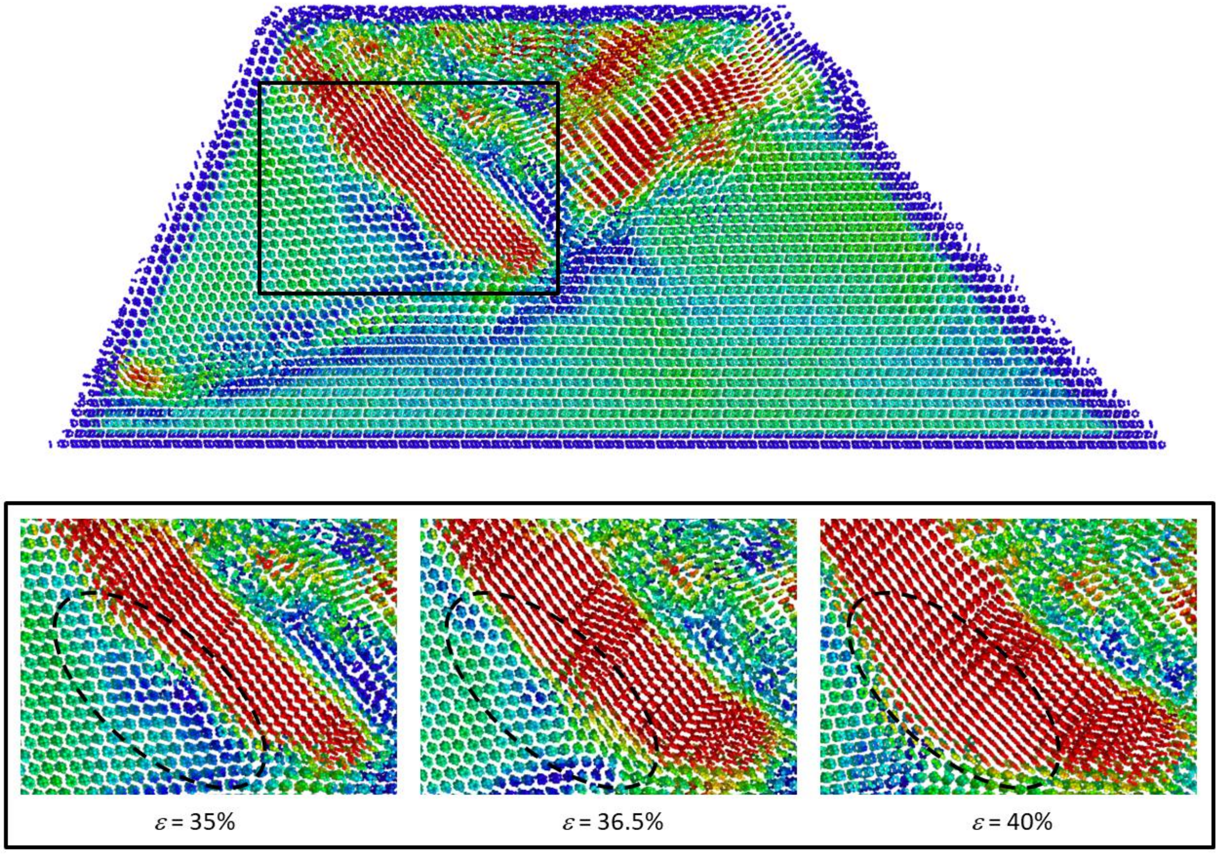

Supplementary Figure 6. Collective re-orientation of benzene rings along the 45° local shear direction. The re-orientation process during compression in the local region in the black box is snapshotted and magnified in the lower zoomed-in images.

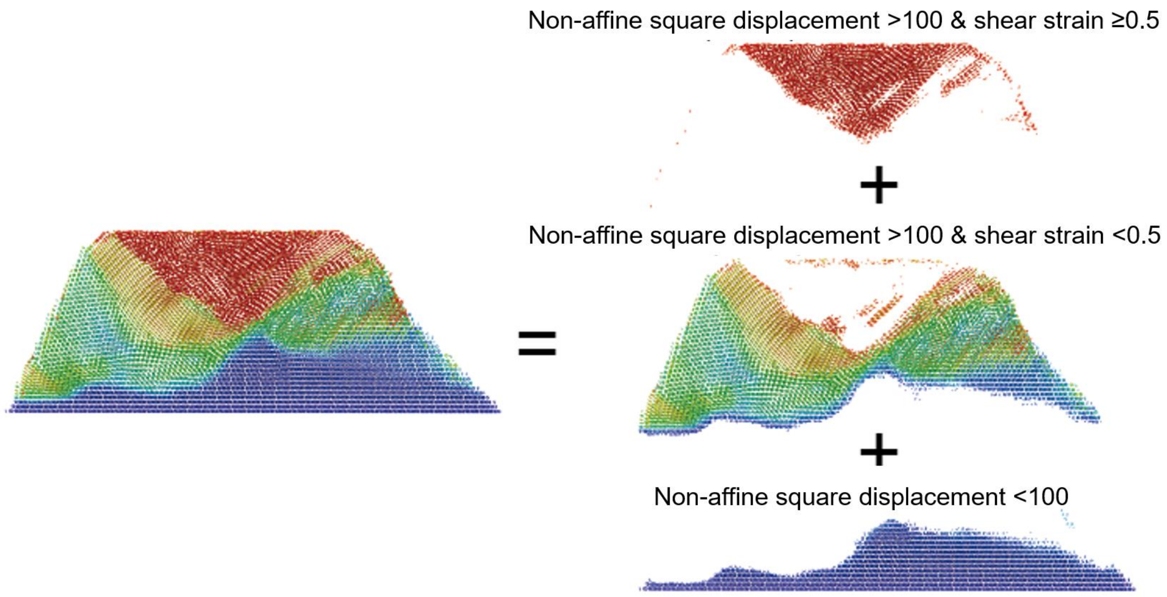

Supplementary Figure 7. Selection of the desification region using parameters of non-affine square displacement >100 and shear strain <0.5 set in software OVITO.

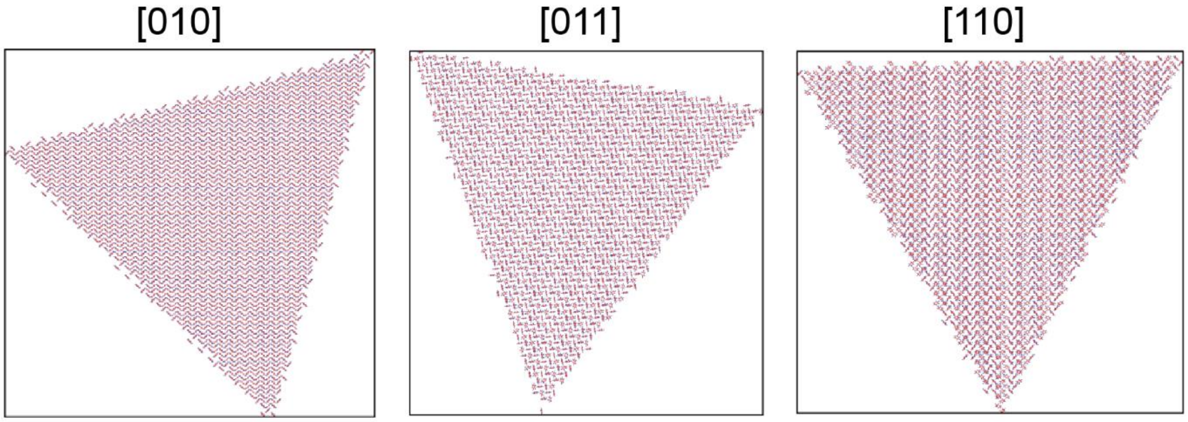

Supplementary Figure 8. The benzene molecule orientation in the lateral face of the pyramidal sample for simulations under different loading directions.

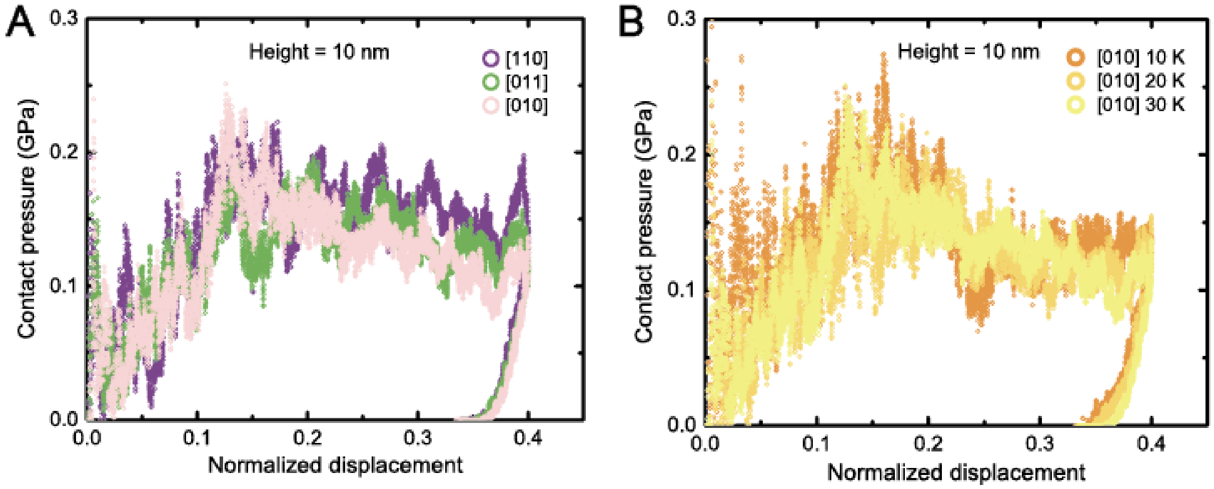

Supplementary Figure 9. Simulated contact pressure-normalized displacement relations using different (**A**) unit cell orientations and (**B**) temperatures in the range of 10-30 K.

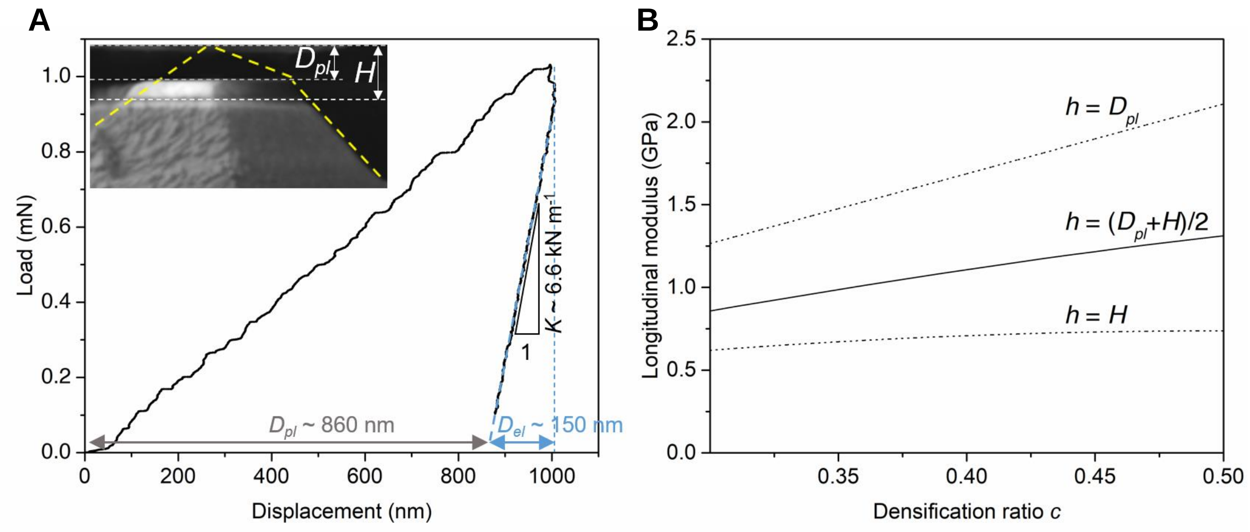

Supplementary Figure 10. Estimation of the longitudinal modulus of densified region: (**A**) Extracting D_{pl}, H, k, and c from compression data (inset: post-mortem SEM image defining the dimensions); (**B**) Bounds of modulus as a function of densification ratio.

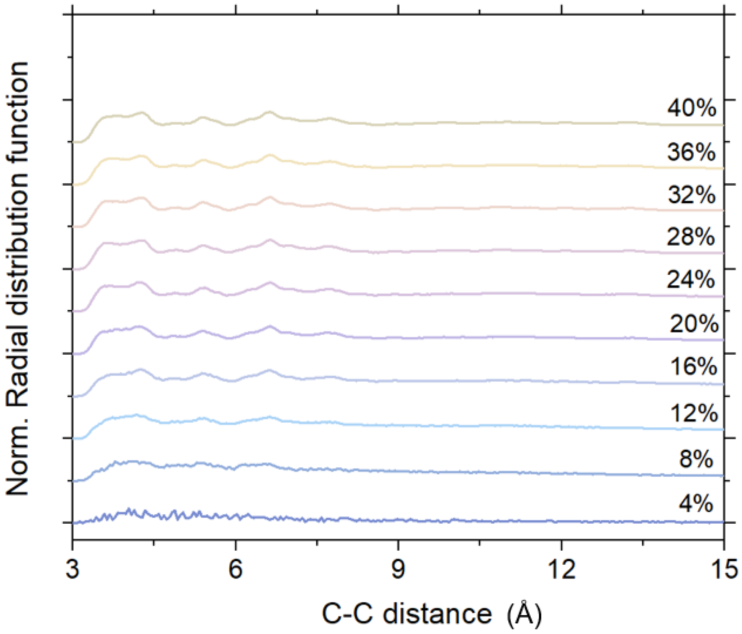

Supplementary Figure 11. Radial distribution function in the region with high shear strain extracted from MD simulation using parameters of non-affine square displacement >100 and shear strain ≥0.5 set in software OVITO.
