# Supplementary material for: Deformation characteristics of solid-state benzene as a step towards understanding planetary geology
Source: Nat Commun. 2022 Dec 26;13:7949. doi: 10.1038/s41467-022-35647-x (PMC9792550; doi:10.1038/s41467-022-35647-x)
Supplement: Supplementary file 2 — Description of Additional Supplementary Files [file 41467_2022_35647_MOESM2_ESM.pdf]

Description of Additional Supplementary Files

File Name: Supplementary Movie 1
Description: In-situ SEM mechanical test on a solid benzene pyramid.

File Name: Supplementary Movie 2
Description: The local-density evolution of a cross-sectioanl slice cut from 10-nm height pyramidal benzene.

File Name: Supplementary Movie 3
Description: The local-density evolution of a cross-sectioanl slice cut from 20-nm height pyramidal benzene.

File Name: Supplementary Movie 4
Description: The local-density evolution of a cross-sectioanl slice cut from 30-nm height pyramidal benzene in parallel with force vs. displacement curve.

File Name: Supplementary Movie 5
Description: The shear-strain evolution of a slice sample cut from 30-nm height pyramidal benzene in parallel with contact pressure vs. normalized displacement curve.
